# Development of a Murine Model for Aerosolized Ebolavirus Infection Using a Panel of Recombinant Inbred Mice

**DOI:** 10.3390/v4123468

**Published:** 2012-12-03

**Authors:** Elizabeth E. Zumbrun, Nourtan F. Abdeltawab, Holly A. Bloomfield, Taylor B. Chance, Donald K. Nichols, Paige E. Harrison, Malak Kotb, Aysegul Nalca

**Affiliations:** 1 Center for Aerobiological Sciences, U.S. Army Medical Research Institute of Infectious Diseases (USAMRIID), 1425 Porter Street, Fort Detrick, Maryland 21702, USA; Email: Holly.A.Bloomfield@us.army.mil (H.A.B.); paigeha@pcom.edu (P.E.H); Aysegul.Nalca@us.army.mil (A.N); 2 Department of Molecular Genetics, Biochemistry and Molecular Biology, University of Cincinnati, 231 Albert Sabin Way, Cincinnati, OH 45267, USA; Email: Nourtan.Abdeltawab@uc.edu (N.F.A.); Malak.Kotb@uc.edu (M.K.); 3 Pathology Division, U.S. Army Medical Research Institute of Infectious Diseases (USAMRIID), 1425 Porter Street, Fort Detrick, Maryland 21702, USA; Email: Taylor.Chance@us.army.mil (T.B.C.); Donald.K.Nichols@us.army.mil (D.K.N)

**Keywords:** Ebola, ebolavirus, filovirus, aerosol, mouse, BXD, recombinant inbred

## Abstract

Countering aerosolized filovirus infection is a major priority of biodefense research. Aerosol models of filovirus infection have been developed in knock-out mice, guinea pigs and non-human primates; however, filovirus infection of immunocompetent mice by the aerosol route has not been reported. A murine model of aerosolized filovirus infection in mice should be useful for screening vaccine candidates and therapies. In this study, various strains of wild-type and immunocompromised mice were exposed to aerosolized wild-type (WT) or mouse-adapted (MA) Ebola virus (EBOV). Upon exposure to aerosolized WT-EBOV, BALB/c, C57BL/6 (B6), and DBA/2 (D2) mice were unaffected, but 100% of severe combined immunodeficiency (SCID) and 90% of signal transducers and activators of transcription (Stat1) knock-out (KO) mice became moribund between 7–9 days post-exposure (dpe). Exposure to MA-EBOV caused 15% body weight loss in BALB/c, but all mice recovered. In contrast, 10–30% lethality was observed in B6 and D2 mice exposed to aerosolized MA-EBOV, and 100% of SCID, Stat1 KO, interferon (IFN)-γ KO and Perforin KO mice became moribund between 7–14 dpe. In order to identify wild-type, inbred, mouse strains in which exposure to aerosolized MA-EBOV is uniformly lethal, 60 BXD (C57BL/6 crossed with DBA/2) recombinant inbred (RI) and advanced RI (ARI) mouse strains were exposed to aerosolized MA-EBOV, and monitored for disease severity. A complete spectrum of disease severity was observed. All BXD strains lost weight but many recovered. However, infection was uniformly lethal within 7 to 12 days post-exposure in five BXD strains. Aerosol exposure of these five BXD strains to 10-fold less MA-EBOV resulted in lethality ranging from 0% in two strains to 90–100% lethality in two strains. Analysis of post-mortem tissue from BXD strains that became moribund and were euthanized at the lower dose of MA-EBOV, showed liver damage in all mice as well as lung lesions in two of the three strains. The two BXD strains that exhibited 90–100% mortality, even at a low dose of airborne MA-EBOV will be useful mouse models for testing vaccines and therapies. Additionally, since disease susceptibility is affected by complex genetic traits, a systems genetics approach was used to identify preliminary gene loci modulating disease severity among the panel BXD strains. Preliminary quantitative trait loci (QTLs) were identified that are likely to harbor genes involved in modulating differential susceptibility to Ebola infection.

## 1. Introduction

The family *Filoviridae* includes two genera, *Ebolavirus* and *Marburgvirus* and a third putative genus *Ceuvavirus *[1]. Filoviruses have caused extremely lethal sporadic human outbreaks, primarily in central Africa, and are classified as Category A Bioterrorism Agents by the United States Centers for Disease Control and Prevention. The primary threat of filoviruses, as weapons of bioterrorism, is thought to be as aerosols [2]. Appropriate animal models are therefore needed for discovery and testing of filovirus countermeasures and for gaining a better understanding of the pathogenesis of airborne filovirus infection. Aerosol models have been developed in non-human primates (NHPs) for Sudan virus (SUDV), Ebola virus (EBOV) and Marburg virus (MARV) [3,4,5]. Filovirus infection of NHPs may provide the best representation of the disease process in humans [6]. However, a small animal model, such as the mouse, would best facilitate screening novel countermeasures. Importantly, a mouse model may also help to reveal host factors involved in susceptibility or resistance to aerosolized filovirus infection; information that could lead to new targets for drug development.

Wild-type (WT) filoviruses are likely unable to suppress the type I interferon response of mice, a feature necessary for productive filovirus infection and the reason that mice are resistant to wild-type filovirus infection [7,8]. Lethal filovirus infection of mice has thus only been achieved with WT virus in immunocompromised mice; or, in WT mice through the use of virus adapted to mice by serial passage [7,9]. A mouse model of airborne filovirus infection was recently developed using IFN-α/β KO knock-out mice, and can be used to study infection with WT strains of EBOV and MARV [10]. Infection of immunocompetent mice with MA-EBOV has been thoroughly studied using the intra-peritoneal (IP) route of viral challenge; however, an aerosol route of delivery has not been reported [9,11,12]. 

One goal of this study was to identify WT, immunocompetent mouse strains that are susceptible to aerosol infection with either WT or MA-EBOV, and that most closely match the disease resulting from aerosol exposure of NHPs to EBOV. Because BALB/c mice were previously found to be susceptible to IP infection with MA-EBOV [9,11,12], these mice were exposed to WT-EBOV or MA-EBOV by the aerosol route. SCID, IFN receptor KO, and Stat1 KO mice have been lethally infected with EBOV by the IP route [7,9], and were therefore tested as likely positive controls for susceptibility to EBOV and MA-EBOV by the aerosol route. Subcutaneous (SC) injection of MA-EBOV typically does not result in infection of WT mice, however, it has been shown that Perforin KO mice can be productively infected with MA-EBOV by the SC route [13]. As such, Perforin KO mice were also evaluated as a likely positive control for susceptibility to airborne EBOV and MA-EBOV infection

To identify wild-type mouse strains susceptible to airborne filovirus infection, a panel of recombinant inbred BXD mouse strains were exposed and screened. Additionally, the genetically diverse BXD panel is a powerful tool for systems genetics approaches that allow unbiased discovery of QTLs associated with various phenotypes and diseases [14,15,16,17,18,19,20]. In the case of infectious diseases, BXD panels have been used to identify genetic loci for differential susceptibility to streptococcal sepsis and H5N1 influenza A virus [18,19,21,22]. The BXD panel of RI and ARI mouse strains were generated by a special scheme of crossing of B6 and D2 mice and their progeny leading to accumulation of recombinations in the genome of each strain [17,23]. These strains exhibit a wide spectrum of phenotypes, based on the overall genetic context of each strain. In addition, the ancestral parental strains are fully sequenced and BXD panel strains are densely genotyped with microsatellite markers and single nucleotide polymorphisms (SNPs) [24,25,26]. 

Five BXD mouse strains had uniform lethality upon exposure to aerosolized MA-EBOV. The BXD strain(s) most susceptible to aerosol filovirus infection identified in this report can be used as the basis for the airborne MA-EBOV infection model in mice.

To gain a greater understanding of the disease mechanism and identify therapeutic targets, a systems genetics approach was employed using a set of 60 BXD RI and ARI mice and their ancestral parental strains (C57BL/6 (B6) and DBA/2 (D2)). Genetic and non-genetic factors that modulate differential susceptibility to MA-EBOV were analyzed and the contribution of each of these cofactors evaluated using multivariate analysis. Preliminary QTLs that contain modifier genes that might be modulating differential response to aerosolized MA-EBOV were mapped. Continued work with this systems genetics approach will likely lead to the identification of additional host factors modulating differential susceptibility to lethal filovirus infection, besides the known effects of type 1 interferon response on infection outcomes. 

## 2. Results and Discussion

### 2.1. Exposure of Wild-Type and Immunocompromised Mice to a High Dose of Aerosolized MA-EBOV

**Figure 1:**
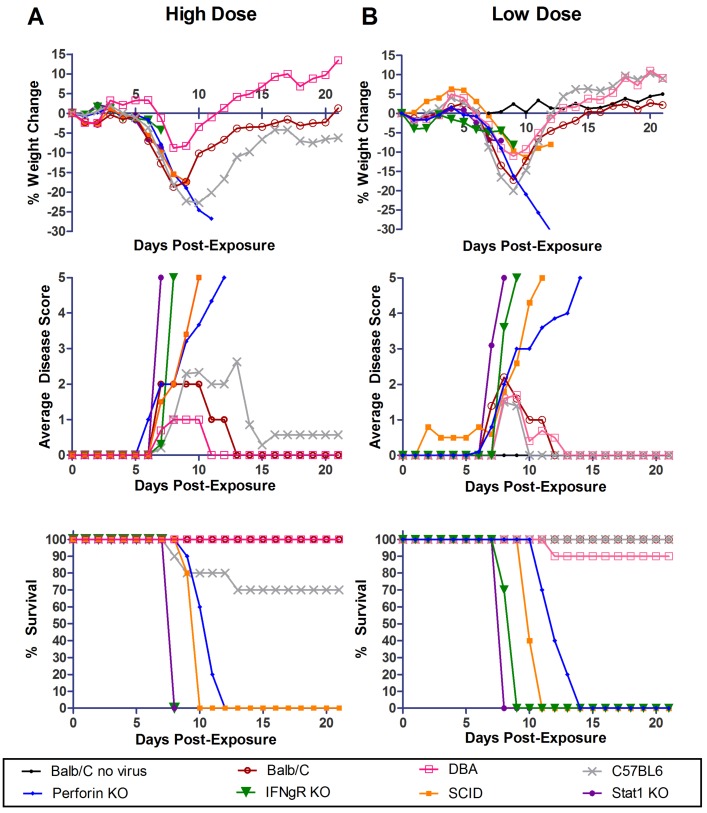
**Exposure of WT and immunocompromised mice to aerosolized WT & MA-EBOV (high dose).** Percent weight loss, clinical disease score and survival of BALB/c, DBA-2, C57BL/6, Perforin KO, IFNγR KO, SCID and Stat1 KO mice are shown for 0-21 dpe (n = 10 per mouse strain) following high 2461 ± 201 pfu (A) or low 228 ± 26 pfu (B) dose aerosol exposures. A BALB/c no virus control was included in the low dose challenge experiment only.

Three WT mouse strains (BALB/c, D2, and B6) and four different immunocompromised mouse strains, Stat1 KO, SCID, IFNγR KO, and Perforin KO, were tested for susceptibility to a high dose of aerosolized MA-EBOV. There were 10 animals of each strain and each animal was exposed to a dose of 2461 ± 201 pfu of aerosolized MA-EBOV. To achieve this “high” dose, undiluted virus stock (2 × 10^7^ pfu/mL) was placed in the nebulizer. All of the strains lost weight (between 5–25%) and showed clinical signs of disease in the form of a general viral prodrome after exposure to virus (Figure 1A). There were no obvious signs of hemorrhage. The onset of clinical signs was 7 dpe for all groups, except signs were apparent at 6 dpe in the Perforin KO mice. All of the BALB/c and D2 mice recovered. Three of ten (33%) B6 mice became moribund between 8–13 dpe, and two of the survivors had permanent neurologic impairment. Exposure to aerosolized MA-EBOV was uniformly lethal in the four immunocompromised strains tested. IFNγR KO and Stat1 KO mice succumbed on 7 dpe whereas SCID and Perforin KO mice became moribund at an average of 9.8 and 10.7 dpe respectively. Overall, mice of each strain became moribund slightly sooner after this high dose exposure, than was observed after exposure to a 228 ± 26 pfu dose (Figure 1B), although this difference was not statistically significant. Following the low dose exposure, no mice B6 became moribund, however, one of 10 D2 mice succumbed to the infection on day 12.

### 2.2. Differential Susceptibility of BXD RI and ARI Mice Strains Exposed to a High Dose of Aerosolized MA-EBOV

Since three standard strains of mice, BALB/c, D2, and B6, were not uniformly susceptible to aerosolized MA-EBOV, even at a high dose, none of these strains were promising as a disease model. Although each of the four strains of immunocompromised mice tested were highly susceptible and could prove useful as models for studying some aspects of MA-EBOV infection, an animal model with an intact immune system is most desirable. Therefore, a panel of 60 BXD RI & ARI mouse strains, which are a genetically diverse pool of strains derived from crossing of B6 and D2 strains, were evaluated for susceptibility to infection with aerosolized MA-EBOV. Additionally, we expected that the diversity of the BXD panel would help to identify a suitable, uniformly susceptible strain for animal model development. 

**Figure 2:**
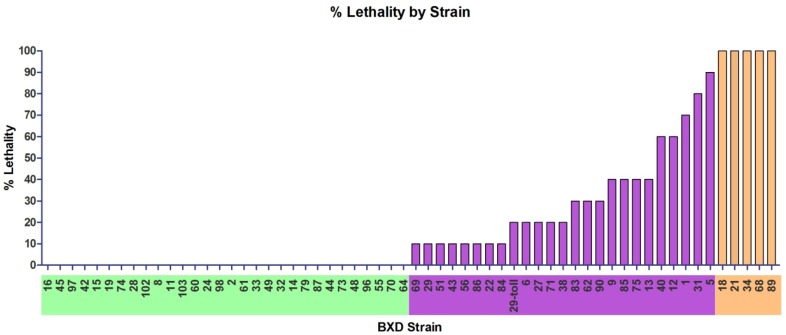
**A range of susceptibility among BXD strains exposed to a high dose of aerosolized MA-EBOV.** BXD strains fell into three groups based on the percent lethality following exposure to 1608 ± 549 pfu airborne MA-EBOV: resistant (green), intermediate susceptibility (purple) and susceptible (orange). Within each group, strains were ordered by survival (if applicable), percent of animals displaying clinical signs and percent weight loss corresponding to Table 1.

Groups of up to 10 female mice of each strain were exposed to an average dose of 1608 ± 549 pfu of aerosolized MA-EBOV. Mice displayed differential susceptibility to aerosolized MA-EBOV, as manifested by the differences in lethality across the strains (Figure 2). The number of mice, average age, average baseline body weight, dose, and data on the disease course is presented for each strain (Table 1). There were differences across the BXD strains in weight loss, survival time and clinical disease scores (Table 1). Of particular importance were stark variations in mortality among the BXD strains with some strains exhibiting uniform lethality. The identification of such strains, which could be used as the basis of models to test novel EBOV therapeutics was a primary aim of our study.

**Table 1:** **BXD strains exposed to a high dose of aerosolized MA-EBOV.** BXD strains fell into three groups based on the percent lethality following exposure to 1608 ± 549 pfu airborne MA-EBOV: resistant (green), intermediate (purple) and susceptible (orange). Within each group, strains were ordered by survival (if applicable), % of animals displaying clinical signs and % weight loss. Data presented are: average age of mice, aerosol dose, average starting weight, maximum % weight loss when all mice from group living, day of onset of clinical signs, % of mice with clinical signs, cumulative disease score, % survival, and first and last days animal became moribund ( n = 10).

BXD Strain	Ave. Age (days)	Dose	Ave. Starting Weight (g)	Max Weight Loss (%)	Onset clinical signs	% with clinical signs	Cumulative disease score	% Survival	1st day Mori-bund	Last Day Mori-bund
**C57Bl6**	44	2573	19.7	-18.1	7	100	169	70	9	13
**DBA**	44	2132	15.2	-8.8	7	100	37	100	na	na
**16**	55	1291	21.2	-9.2	na	0	0	100	na	na
**45**	63	301	17.8	-11.3	na	0	0	100	na	na
**97**	55	1244	17.4	-11.7	na	0	0	100	na	na
**42**	70	1093	19.5	-12.8	na	0	0	100	na	na
**15**	55	1335	22	-13	na	0	0	100	na	na
**19**	51	2295	17.5	-14.6	na	0	0	100	na	na
**74**	41	1335	15	-14.7	na	0	0	100	na	na
**28**	78	1808	22.8	-23.7	na	0	0	100	na	na
**102**	71	2782	19.4	-13.5	8	10	1	100	na	na
**8**	78	1466	20	-19.6	6	10	2	100	na	na
**11**	55	1145	17.9	-11.3	9	50	8	100	na	na
**103**	63	2650	18.2	-18.6	8	50	9	100	na	na
**60**	70	1562	20.6	-19.7	9	80	16	100	na	na
**24**	49	1721	17.2	-9	7	100	64	100	na	na
**98**	57	1126	16	-11.2	7	100	13	100	na	na
**2**	55	1356	22.4	-11.8	7	100	12	100	na	na
**61**	63	1279	19.9	-14.8	7	100	41	100	na	na
**33**	63	1667	15.5	-16	8	100	20	100	na	na
**49**	67	1632	21.8	-16.1	8	100	15	100	na	na
**32**	71	1910	21.1	-17.2	8	100	26	100	na	na
**14**	61	1577	21.7	-17.4	8	100	15	100	na	na
**79**	66	2169	18.7	-18.2	8	100	14	100	na	na
**87**	56	279	16.1	-18.7	9	100	21	100	na	na
**44**	55	949	16.2	-18.9	7	100	40	100	na	na
**73**	51	2369	17.8	-20.1	7	100	35	100	na	na
**48**	77	1655	22.2	-20.5	8	100	24	100	na	na
**96**	70	1592	24.2	-20.5	7	100	30	100	na	na
**55**	70	1280	19.9	-20.6	7	100	26	100	na	na
**70**	70	2409	21.5	-21	6	100	38	100	na	na
**64**	46	1505	17.6	-24.7	6	100	40	100	na	na
**69**	63	1557	18.4	-13.4	7	100	56	90	11	11
**29**	64	1600	19.3	-13.5	9	50	10	90	10	10
**51**	62	1584	21	-16.7	6	100	28	90	17	17
**43**	75	1777	19.1	-17.7	9	100	20	90	17	17
**56**	65	1285	20	-18	20	10	5	90	20	20
**86**	80	343	21.2	-18.7	9	100	24	90	10	10
**22**	70	964	16.5	-18.9	8	100	53	90	10	10
**84**	68	2212	19.2	-20.3	6	100	42	90	13	13
**29-Toll**	74	1564	18.8	-11.6	8	100	30	80	10	11
**6**	58	1141	17.8	-15	8	100	26	80	9	10
**27**	51	2503	19.6	-16.3	7	100	51	80	10	11
**71**	68	1303	20.4	-20.3	6	100	68	80	12	14
**38**	65	2355	17.6	-21	7	100	98	80	12	12
**83**	60	2728	18.9	-12.3	7	100	49	70	9	10
**62**	77	2233	19.4	-16.6	9	100	45	70	9	11
**90**	53	1170	16.8	-17	7	100	70	70	10	10
**9**	70	1492	30	-17.8	7	100	90	60	11	123
**85**	58	1347	19.4	-18.2	5	100	107	60	10	14
**75**	49	1575	18.7	-19.4	8	100	54	60	10	12
**13**	54	1249	20.3	-19.8	9	100	37	60	10	13
**40**	75	1783	19.2	-17.3	8	100	92	40	11	11
**12**	65	1101	17.2	-20.4	7	100	99	40	9	10
**1**	60	1221	19.5	-21	7	100	117	30	8	12
**31**	71	1735	21.5	-14.5	8	100	84	20	8	11
**5**	68	1429	19	-16.9	8	100	136	10	10	17
**18**	78	1552	21.3	-17	8	100	95	0	9	12
**21**	51	2036	14.9	-17.7	7	100	103	0	10	11
**34**	78	2110	24.1	-11.97	6	100	81	0	7	7
**68**	51	2408	18.3	-20.2	7	100	90	0	10	10
**89**	47	1345	20.3	-13	7	100	93	0	9	10

### 2.3. Uniform Lethality in Five BXD Mouse Strains Exposed to a High Dose of Aerosolized MA-EBOV

**Figure 3:**
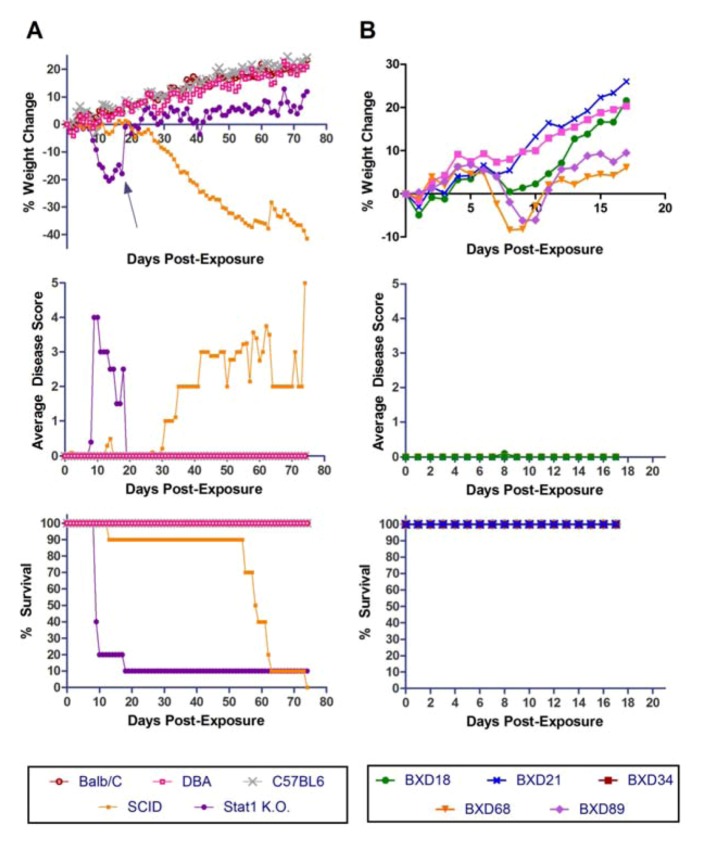
**Exposure of susceptible BXD strains to high (a) and low (b) doses of aerosolized MA-EBOV.** Weight loss, clinical disease score and survival are shown for BXD18, BXD21, BXD34, and BXD68 exposed to high (1345-2408 pfu) or low (52 pfu) doses. BXD89 was exposed to a high dose only (n = 10 per mouse strain).

Figure 3A shows the weight, clinical disease scores and survival of only the five BXD mouse strains for which a high dose of aerosolized MA-EBOV infection resulted in a uniformly lethal disease. The actual dose delivered to these mice ranged from 1345–2408 pfu. Moribund condition occurred between days 7–12 and weight loss was ≥15%. 

### 2.4. Exposure of Susceptible BXD Mouse Strains to a Low Dose of Aerosolized MA-EBOV

For comparison purposes, four of five BXD strains with known 100% susceptibility to a high dose of MA-EBOV were tested for susceptibility to a low-dose (an average of 52 pfu) of MA-EBOV by the aerosol route. BXD89 was not available for this test. The onset of clinical disease scores in all strains was comparable to that seen with the high dose exposure and all strains had a weight loss of ≥10% (Figure 3B). However, lethality ranged from 0% (for BXD18) to 100% at the low dose with only one strain (BXD34), and another with 90% lethality (BXD68). BXD21 had 10% lethality. These data suggests that two BXD strains, BXD34 and BXD68, are the most susceptible to aerosolized MA-EBOV of the 60 BXD strains tested and will be used for further experiments. 

### 2.5. Tissue Viral Load and Histopathology in BXD Mouse Strains Exposed to Aerosolized MA-EBOV

**Figure 4:**
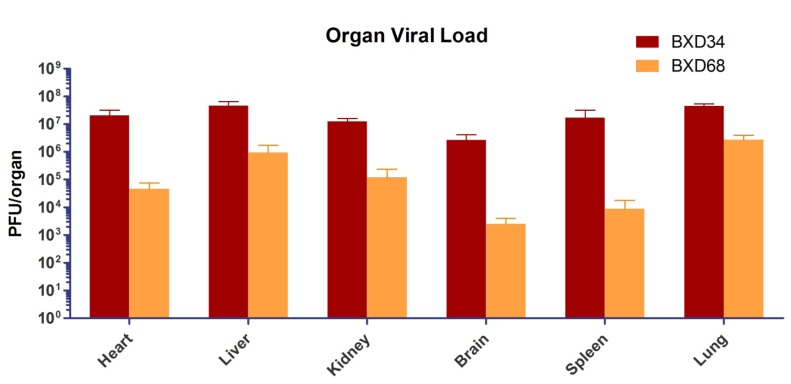
Average organ viral loads for the two most susceptible BXD strains, BXD34 (n = 5) and BXD68 (n = 4) after aerosol exposure to MA-EBOV. Organs were taken after humane euthanasia of moribund mice (7–9 dpe). The pfu/tissue represents the viral load within the entire organ, since the entire organ was homogenized for each data-point.

All mice that became moribund after low dose exposure to airborne MA-EBOV were necropsied. The organs were placed in fixative for histopathology analysis (n = 5 for BXD34 and BXD68 or titrated by plaque assay for viral load (n = 5 for BXD34 and n = 4 for BXD68). Heart, liver, kidney, brain, spleen and lung were evaluated for viral load by plaque assay (Figure 4). Tissue viral loads ranged from 2.5 × 10^3^ pfu to 4.5 × 10^7^ pfu per organ. More MA-EBOV was detected in tissues from BXD34 mice than BXD68 for each organ measured. Mice from both groups were exposed at the same time and received the approximately same dose of MA-EBOV. The increased organ viral loads in BXD34 ranged from 10 to15-fold more for lung and liver, approximately 250-fold more in the heart, and >1000-fold more in the brain and spleen. 

### 2.6. Histological Findings

**Figure 5:**
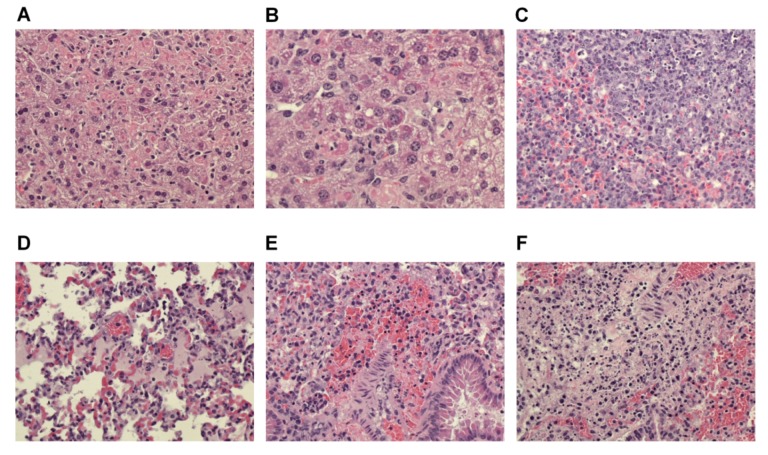
Histopathology in tissues of most susceptible BXD strains after aerosol exposure to a low dose of MA-EBOV. Hematoxylin and eosin (H&E) stained tissues were examined from moribund BXD34 (n = 5) and BXD68 (n = 5) mice. Representative images are shown. (**a**) Section of liver with multiple pale-staining necrotic hepatocytes accompanied by infiltrates of occasional neutrophils. (**b**) Higher magnification of liver showing that, adjacent to areas of necrosis, many hepatocytes contain one or more dark-red intracytoplasmic inclusion bodies. (**c**) Spleen with foci of necrosis in the red pulp (lower left) and white pulp (upper right). Photos D-F are from strain BXD68 lung. (**d**) Pulmonary congestion and edema with scattered necrotic cells within alveolar lumina and lining alveoli. (**e**) Higher magnification showing necrosis of alveolar septa with hemorrhage into the small airways. (**f**) Focus of more extensive alveolar septal necrosis with hemorrhage and low numbers of neutrophils.

Similar to previous studies of EBOV in mice, none of the susceptible BXD mice had evidence of systemic hemorrhagic diathesis or disseminated intravascular coagulation (DIC), which can variably occur with EBOV infection in humans and nonhuman primates (NHPs). However, all of the susceptible BXD mice analyzed had hepatic lesions consistent with those that have been previously reported in mice and NHPs with fatal systemic ebolavirus infection (Table 2). These lesions consisted of multiple, random foci of hepatocellular degeneration and necrosis with few associated inflammatory cells (usually neutrophils) (Figure 5A). Within hepatocytes adjacent to areas of necrosis in BXD34 mice, there were numerous eosinophilic intracytoplasmic inclusion bodies characteristic of infection with ebolavirus (Figure 5B). Virus-induced intracytoplasmic inclusion bodies were detected within hepatocytes of all BXD34 mice but not in BXD68 mice. Similar hepatic inclusions have been seen in NHPs and in mice, but are not consistent findings [7,12,27]. The presence of these inclusions in all five mice from the BXD34 group and in none of the five animals from the BXD68 group could possibly be related to the mouse genetic strain, viral dose, and/or the fact that the BXD34 mice became moribund 1–2 days earlier than the BXD68 mice.

**Table 2:** Summary of significant histopathologic findings.

Strain	Ave.Dose (pfu)	Day p.e.	Liver*	Spleen	Mesenteric LN	Lungs	Adrenals
BXD34	53	8	lesions	necrosis	lymphocytolysis	WNL**	cortical necrosis
BXD68	51	9	lesions	necrosis	lymphocytolysis	mf*** necrosis	cortical necrosis

p.e. = post-exposure * Typical hepatic lesions of ebolavirus infection in mice are described in the text ** WNL = within normal limits *** mf = multifocal

Within the spleen of all susceptible BXD mice, there was mild to moderate necrosis of the red and white pulp with no associated inflammatory cells (Figure 5C). Similar to the white pulp of the spleen, there was lymphocyte necrosis in lymphoid follicles that ranged in severity from minimal to mild. Many of the necrotic lymphocytes were phagocytized by macrophages, forming tingible-body macrophages. Lymphocyte death in the mandibular and/or the bronchial/mediastinal lymph nodes was also observed. In most of the susceptible BXD mice, there was mild to moderate lymphocyte necrosis throughout the thymic cortex. Within the adrenal glands, there was minimal to mild multifocal necrosis of cortical cells along the corticomedullary junction in all of the BXD68 mice, three of five BXD34 mice analyzed. 

In all five BXD68 mice, there was mild to moderate multifocal congestion and edema of lung lobes with necrosis of cells lining alveoli and within alveolar lumina (Figure 5D–F). There were few neutrophils associated with necrotic lesions. There were no lung lesions present in BXD34 mice.

The lesions present in the livers, spleens, and adrenal glands of BXD mice exposed to aerosolized MA-EBOV are consistent with those previously reported in mice with fatal, systemic MA-EBOV infection after intraperitoneal (IP) viral inoculation [11,12,27]. The lymphocyte death in the thymus, mesenteric lymph node, and other lymph nodes in these mice is also a common lesion reported to be associated with systemic MA-EBOVs infection. However, necrosis of lymphocytes in mice is also a common, nonspecific response to stress and/or inflammation. 

### 2.7. Susceptibility of WT and Immunocompromised Mouse Strains to Aerosolized WT-EBOV

**Figure 6:**
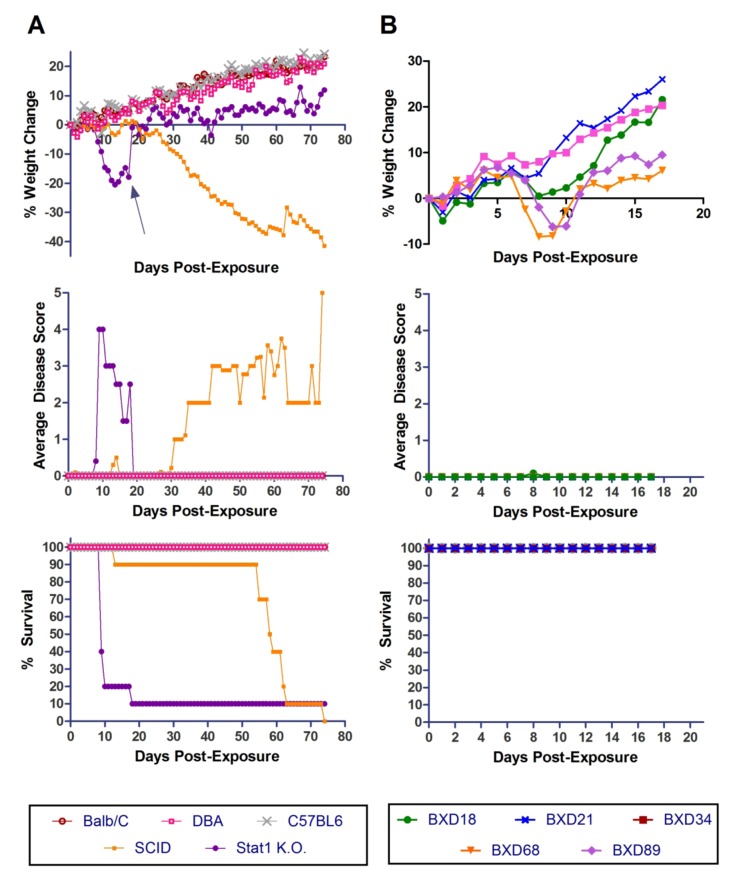
**Exposure of mouse strains to aerosolized WT-EBOV.** (**a**) BALB/c, DBA/2, C57BL/6, SCID and Stat1 KO mice and (**b**) BXD18, BXD21, BXD34, and BXD68 and BXD89 mice were exposed to non-adapted EBOV by the aerosol route and monitored for % weight change, disease score and survival for 17–80 days (n = 10 per mouse strain).

Three WT mouse strains (BALB/c, B6 and D2) and two immunocompromised/ knock-out mice (Stat1 KO and SCID) were tested for susceptibility to aerosolized WT-EBOV. None of the WT mouse strains lost weight or showed clinical signs of disease (Figure 6A). In contrast, exposure to aerosolized WT-EBOV was uniformly lethal in SCID mice and 90% lethal in Stat 1 knockout mice. The time-to-death or euthanasia in these two strains was greater for WT-EBOV aerosols than was observed after exposure to aerosolized MA-EBOV. Interestingly, a moribund condition took much longer to develop in SCID mice exposed to aerosolized WT-EBOV than Stat 1 KO mice.

### 2.8. Susceptibility of BXD Strains to Aerosolized WT-EBOV

The five BXD recombinant inbred strains (Jackson Laboratories) that were found to be most susceptible to aerosolized MA-EBOV were tested for susceptibility to aerosolized WT-EBOV (Figure 6B). Mice were exposed to an average dose of 392 pfu and were weighed and scored daily. Of the five strains tested, two lost a significant amount of weight. However, no mice succumbed in any group and no mice from any of the groups displayed any signs of illness (scores were 0 for all mice on all days except for one mouse on one day, which received a score of "1"). Therefore, BXD mice that are susceptible to airborne infection with MA-EBOV are not susceptible to WT-EBOV at the dose given. However, it is possible that increased disease and/or lethality could be observed at a higher dose. 

### 2.9. Multiple Covariate Analysis Confirms the Role of BXD Mouse Strains Genetic Background in Modulating Differential Susceptibility to Aerosolized MA-EBOV

The scheme of generating these BXD strains allowed for accumulation of recombinations followed by inbreeding, therefore the genetic context of each strain is unique and fully inbred. Accordingly, each unique strain is represented by an infinite number of mice, and this contributes to the observed high reproducibility, and to the strength and power of statistical analyses. These features are beneficial for discovering QTLs in an unbiased manner, and for analyzing genetic loci associated with differential susceptibility to diseases, in this case aerosolized MA-EBOV infection. 

**Figure 7:**
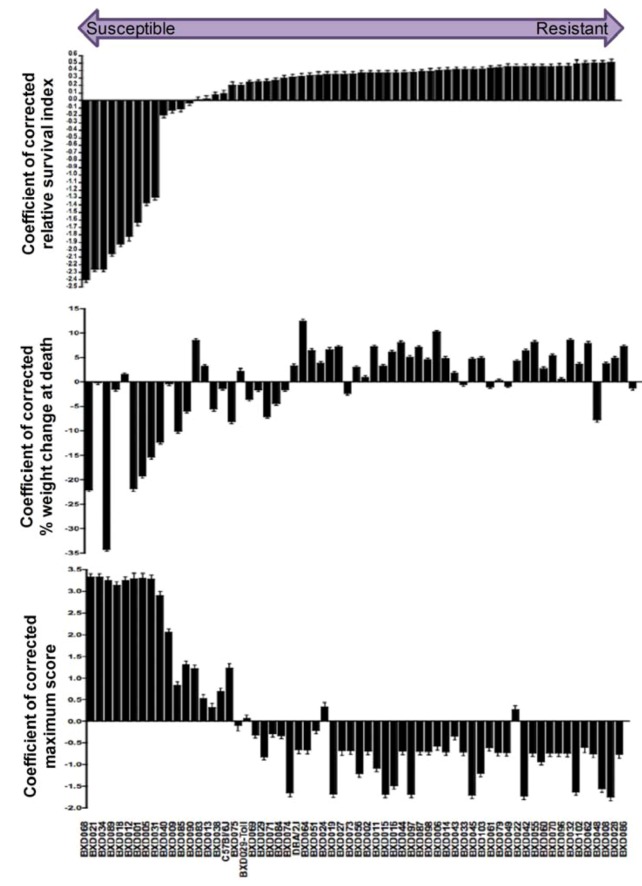
Differential susceptibility of recombinant and advanced recombinant inbred BXD strains & their parental strains post-exposure to aerosolized MA-EBOV. Rank-ordered bar chart of differential survival of 60 BXD strains and their parental strains (n= 586 mice) expressed as coefficient of mean corrected relative survival index. Top panel: Strains are arranged from highly susceptible on the left-hand side to intermediate and finally highly resistant on right-hand side. Middle Panel: Variation in percent of body weight loss at death. Bar chart showing coefficient of mean corrected percent of body weight loss at death arranged in order of differential survival. Bottom Panel: Variation in maximum clinical disease score. Bar chart shows coefficient of mean corrected maximum score arranged in order of differential survival. Error bars represent standard error of the coefficient. Total number of mice used per strain is indicated in Table S1 (Supplemental Information). Statistical test used is two-way ANOVA with correction to covariates as detailed in methods section.

Having ruled out contributions of confounding factors on the observed differential susceptibility to MA-EBOV (see Supplemental Information), interactions between covariates and the genetic factors were determined. First, a “relative survival index,” which is an ordinal scale for mouse survival, irrelevant of its strain, was generated to normalize survival across experiments, and to minimize the inevitable experiment-to-experiment variations. To do so, survival days were graphed as dot plots and the distribution of survival days among BXD strains used within each experiment was determined. A multimodal distribution of survival days fell into three clusters, susceptible, intermediate, and resistant. A survival index was assigned to each mouse according to which cluster it fell within. Survival indices ranged from 0.25–1, 1.25–2, and 2.25–3 for susceptible, intermediate, and resistant clusters respectively. Survival indices were then subjected to 2-way ANOVA using general linear model (GLM) analyses to generate “Coefficient of corrected relative survival index” (Supplemental Information). This coefficient is a mean survival index for each strain, expressing the contribution of each BXD strain on survival taking into account confounding cofactors (age and weight) that might affect differential response (Figure 7). Using GLM analysis of covariates, the relative effect of each covariate (including mouse strain) on survival was determined. Mouse age was not a significant covariate modulating differential response; whereas mouse strain was the most significant predictor of survival (*p* ≤ 0.0001) (Supplemental Information).

### 2.10. Preliminary Genome-Wide QTL Mapped Traits Associated with Differential Response of BXD Strains to Aerosolized MA-EBOV

**Figure 8:**
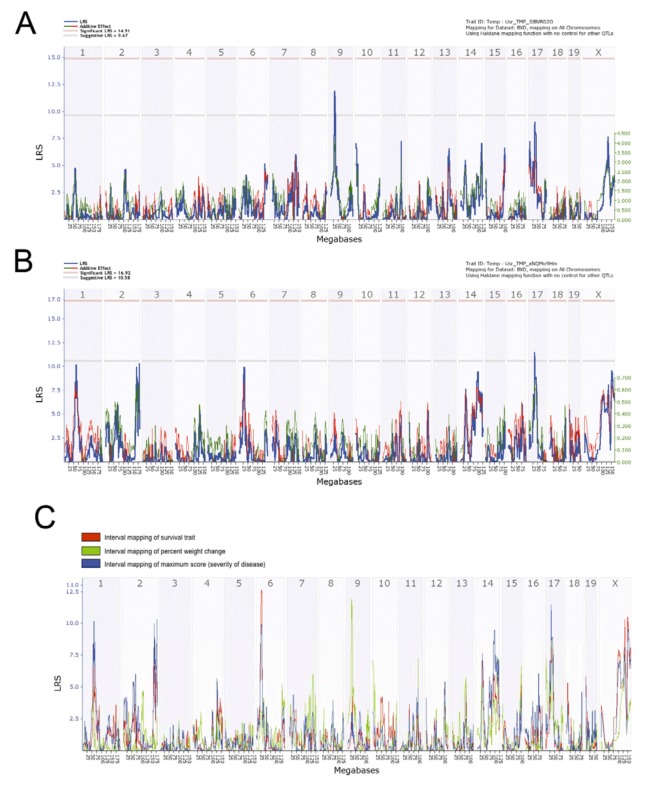
Comparison of preliminary genome-wide QTL mapped traits associated with differential response of BXD strains to aerosolized MA-EBOV. Interval mapping of percent weight loss at death (a) and maximum disease score (b) of BXD strains post-exposure to aerosolized MA-EBOV. Suggestive preliminarily QTLs were mapped. Proximal chromosome 9 is associated with percent weight loss at death (a) and proximal Chromosome 17 is associated with maximum disease score (b). (c) Preliminary QTL comparison of the three mapped traits associated with differential response to EBOV across BXD strains used. Chromosomes 17 and X are preliminary QTLs common for all three traits; chromosome 6 is most significant and associated with both differential survival and maximum disease score; chromosome 9 is associated with percent weight loss at death. Mouse chromosomes are depicted on the upper X-axis, and a physical map of each chromosome on lower X-axis (expressed in mega bases). The Y-axis shows significance of phenotype to genotype association expressed as LRS computed by WebQTL suite of GeneNetwork platform using 2000 permutation tests.

WebQTL, the mapping suite of the GeneNetwork (GN) platform was used to perform interval mapping of traits associated with differential response to high dose of aerosolized MA-EBOV. Each of the traits (phenotypes) associated with differential response is analyzed in the context of genotypic variations of BXD strains. Significance of phenotype-genotype association is assessed by 2000 permutation tests and expressed in likelihood ratio statistics (LRS). Phenotype-genotype linkage mapping of the BXD differential response traits to MA-EBOV showed preliminarily QTLs on chromosomes 6, 9, 17 and X (Figure 8 and Supplementary Information). General haplotype analysis of strains at the mapped QTL on chromosome 6 showed this region has genomic recombinations among the BXD strains, and this will allow to fine-tune and narrow down the QTL by including additional strains, based on genomic variation within the mapped interval (Figure 8 and Supplementary Information). 

Suggestive preliminarily QTLs were mapped on proximal chromosome 9, associated with percent weight loss at death (Figure 8A), and on proximal chromosome 17, associated with maximum disease score (Figure 8B). These QTLs harbor polymorphic genes that are currently being investigated further. Contribution of more than one QTL to the disease outcome is not unusual as it reflects complex trait diseases. To further analyze the contribution of each QTL to disease outcome, we performed QTL comparison of the preliminary three mapped traits associated with differential response to MA-EBOV across BXD strains used (Figure 8C). The QTL on proximal chromosome 6 had the highest LRS (although not reaching significance) and it is associated with both differential survival and maximum disease score. For all three traits, suggestive QTLs on Chromosome 17 and X were found and these should be further investigated and verified. The suggestive QTL on chromosome 9 was associated with one trait only, namely percent weight loss at death. Further experiments to decipher the role of each of these QTLs in modulating differential response to MA-EBOV should shed light as to disease pathogenesis and the mechanisms underlying differential severity.

### 2.11. Linkage Analysis and Haplotype Mapping of Preliminary Survival QTLs Associated with Susceptibility to Airborne MA-EBOV.

**Figure 9:**
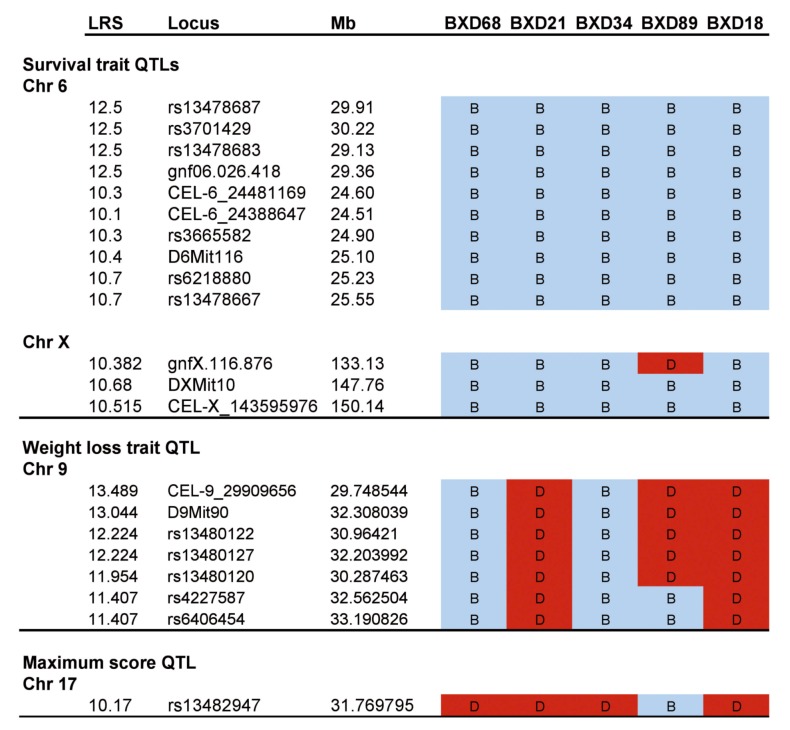
**Linkage analysis and haplotype mapping of preliminary survival QTLs. **(**a**) Linkage analysis of QTLs associated with differential response post-exposure to aerosolized MA-EBOV. Linkage analysis of highest LRS of QTLs associated with differential survival, percent weight loss and maximum score. For each mapped trait, haplotypes of BXD strains showing highest lethality post-exposure to MA-EBOV (BXD68, BXD21, BXD34, BXD89 and BXD18) are analyzed. (**b**) Strain distribution patterns (SDP) and haplotype map of all available BXD strains at the most promising QTL mapped on chromosome 6. “B” = allele from C57BL/6 and “D” = allele from DBA/2 .

Considering that one of the main aims of this study is to select strains that could be used to test therapeutic interventions, we were especially interested in deciphering the genetic background of the five BXD strains that showed marked lethality post-exposure to high dose MA-EBOV. Therefore, haplotype analyses of these five BXD strains, focusing on QTLs associated with differential response to aerosolized MA-EBOV were performed. Using Linkage analyses, QTLs were chosen with LRS higher than 10 and thus are likely to harbor genes that modulate differential survival, weight loss, and maximum disease score (Figure 9). The highest LRS associated with differential survival was located on proximal chromosome 6 between markers CEL-6_24481169 and rs3701429. Analysis of the haplotypes of the five most susceptible BXD strains between these markers showed that they carry the B6 allele (relatively susceptible parental strain) (Figure 9). In addition, linkage analyses revealed that these five strains have a mix of B6 and D2 alleles at the other preliminary QTLs, except for BXD68 and BXD34, which were the most susceptible strains. Further challenge of these QTLs to determine genes and pathways that might be modulating differential response to EBOV will be done in future experiments.

## 3. Experimental Section

### 3.1. Mice

Female mice, aged 6–12 weeks, were allowed to acclimate to the bio-safety level (BSL) 4 suite for 1 week before exposure. BALB/c, C57BL/6J (B6), DBA/2 (D2) and SCID (BALB/c background) mice were purchased from The National Cancer Institute (Frederick, MD). Stat1 KO (129S6/SvEvTac background) mice were purchased from Taconic (Rockville, MD). IFNγR KO (C57BL/6 background), Perforin KO (C57BL/6 and BALB/c background), and BXD mice were purchased from Jackson Laboratories (Bar Harbor, Maine). Sixty different BXD strains were used as well as the B6 and D2 parental strains (n = 600 (10 per group infected), n = 586 mice included in genetic analysis; belonging to the panels of recombinant inbred (RI) and advanced recombinant inbred (ARI) strains, which were generated as previously detailed [24,25,26]. ^24−26^. 

RI strains (BXD1-BXD42) were generated by Taylor *et al. *at Jackson laboratories, by crossing of B6 and D2 mice generating F1 hybrids, which were crossed to generate F2 progeny, each with random patterns of recombination [26] ^26^. F2 hybrids were assigned as parental for strains and sibling mated to generate inbred RI strains. ARI strains (BXD43 and higher), however, follow a scheme of crossing of F2 mice to generate F3 and so on, through F11. F14 or F19 generations were inbred by sibling mating for >20 generations to achieve homozygosity. This breeding scheme was done to increase recombination events resulting in significantly more recombinations per strain compared to conventional RI strains [15,23]. 

The genomes of the B6 and D2 parental strains have been sequenced and a database of their SNPs is available at the Gene Network (GN) web site [28]. Simple sequence length polymorphism (SSLP) markers were typed for all BXD RI strains as previously described [23]. The BXD progeny is genotyped at 13377 SNPs and microsatellite markers [23], a selected subset of 3795 SNPs and microsatellite markers used by GN BXD genotype dataset for mapping traits, can be downloaded [29]. 

### 3.2. Ethics and Animal Welfare

Research was conducted in compliance with the Animal Welfare Act and other federal statutes and regulations relating to animals and experiments involving animals, and adhered to the principles stated in the Guide for the Care and Use of Laboratory Animals (National Research Council, 2011). The facility where this research was conducted (USAMRIID) is fully accredited by the Association for the Assessment and Accreditation of Laboratory Animal Care International (Frederick, MD). Research was conducted under a protocol approved by the Institutional Animal Care and Use Committee (IACUC) at USAMRIID. All animals were observed daily as described below in the “Post-exposure Monitoring” section of these methods. Early endpoint criteria, as also described in the “Post-exposure Monitoring” section below, were used to determine when animals should be humanely euthanized.

### 3.3. Virus

MA-EBOV and EBOV were diluted to an appropriate concentration for aerosol exposure into the three-jet Collision nebulizer (9 x 10^6^ pfu/mL for EBOV, 1x10^7^ pfu/mL and 1 × 10^6^ pfu/mL for the low and high doses of MA-EBOV) in Eagle's Minimum Essential medium (EMEM) (Gibco) containing 10% fetal bovine serum. 

### 3.4. Aerosol Exposures

Mice were transferred to wire mesh cages (up to 10 mice per cage) and up to four wire mesh cages were placed in a whole-body aerosol chamber within a class three biological safety cabinet located inside a BSL-4 suite. Mice were exposed to aerosolized EBOV or MA-EBOV created by a three-jet collision nebulizer for 10 min at a constant flow rate of 19 L/min. The particle sizes generated had a mass median aerodynamic diameter (MMAD) of 1.2–1.8 µm and a geometric standard deviation (GSD) of 1.8-2.1, as measured by an Aerodynamic particle sizer (TSI Incorporated, Shoreview, MN) (data not shown). Relative humidity reached a steady state of 43-65% and temperature was ambient (approximately 20-22 °). Samples were collected from the all-glass impinger (AGI) sampling at a rate of 6 L/min connected to the aerosol chamber and were analyzed by performing plaque assays to determine the inhaled dose of EBOV or MA-EBOV. The dose was calculated using the following formulas: Dose = [Aerosol] (µg/mL) × minute volume (mL) × exposure time (min); minute volume = 2.1(weight (g)) °^.75^. 

### 3.5. Post-Exposure Monitoring

Mice were observed at least once daily beginning the day of exposure and continuing for at least 21 days or until an animal met the clinical criteria for euthanasia. During the daily observations, temperature and body weight were assayed and each mouse was monitored for clinical signs of infection or unexpected illness. Clinical disease scores were assigned based on grooming and provoked behavior, ranging from 0 for no illness, to 5 for severe illness/moribund condition. Scores were defined as: 0 = normal; 1 = reduced grooming, subdued behavior but normal when stimulated; 2 = dull/rough coat, subdued behavior even when stimulated; 3 = absence of grooming/hunched posture; 5 = unresponsive when stimulated, weak/unable to walk. Animals that met the clinical criteria (a score of 5, as defined in the approved research protocol) were humanely euthanized. A full necropsy was then performed on some mice, with collection of tissue samples for analysis of histopathology or virus content from subsets of animals.

### 3.6. Plaque Assay

Assessments of viral concentration were performed on samples from the starting concentration loaded into the nebulizer, AGI contents, and tissues collected at necropsy. Tissues (entire organ) were first homogenized using a gentleMACS dissociator (Miltenyi Biotec) and brought up to equivalent volumes. All samples were then diluted in EMEM with 10% fetal bovine serum and 100 µl inoculated onto 6-well dishes containing Vero E6 cells (ATCC) at approximately 95% confluence. After a 1 h incubation at 37 °C, wells were overlaid with a 0.5% agarose Basal Medium Eagle with Earle’s Salts (EBME) with HEPES (4-(2-hydroxyethyl)-1-piperazineethanesulfonic acid) solution. On day 7, a secondary agarose overlay containing 5% neutral red was added and plaques were counted the following day. For the nebulizer samples and AGI contents, viral concentrations were calculated as plaque-forming units (pfu) per µl; organ viral loads are reported as pfu/organ. For titering virus in organs the entire organ was taken and brought up to equivalent volumes across all samples. 

### 3.7. Histopathology

The tissue samples collected for histology were fixed by immersion in a container of 10% neutral buffered formalin and held for a minimum of 21 days under BSL-4 containment. The following tissues were then prepared for histologic evaluation: brain, cerebellum, eyes, maxillary structures, pituitary gland, lungs, thymus, heart, trachea, esophagus, thyroid glands, liver, spleen, pancreas, gastrointestinal tract, mesenteric lymph nodes, kidneys, adrenal glands, sex organs (uterus and ovaries), urinary bladder, salivary gland, mandibular lymph node, haired skin, skeletal muscle, and bone (femur). The tissue samples were trimmed, routinely processed, and embedded in paraffin. Sections of the paraffin-embedded tissues, 5 µm thick, were cut for histology. The histology slides were deparaffinized, stained with hematoxylin and eosin (H&E), cover-slipped, and labeled. 

### 3.8. Quantitative Trait Loci (QTL) Mapping

QTL mapping was performed using web-based complex trait analysis available on the GN website (http://www.genenetwork.org) and the mapping module which analyzes phenotypes in context of mouse genotypic differences. Interval mapping evaluates potential QTL at regular intervals and estimates the significance at each location using 2000 permutation tests [30]. WebQTL evaluates potential QTL at regular intervals and estimates the significance at each location with a graphical representation of the likelihood ratio statistics (LRS); where LRS = 4.61 X LOD (log of odds). Mapping was done using three traits representative of differential severity to EBOV exposure, namely; differential survival post-exposure expressed as coefficient of corrected relative survival indices, percent weight loss at death and maximum disease score. 

### 3.9. Data Handling and Statistical Analysis

All preliminary data sorting and calculations were performed using features and functions of Microsoft Excel. Data from Excel files were later exported to Data Desk (version 6.2.1) software (Data Description, Inc., Ithaca, NY. DataDesk software was used for all statistical analyses including index clustering, transformations, correlation analysis, linear regression, and general linear model (GLM) analysis. Covariates association was evaluated for significant associations by Spearman correlation as described previously ^18^. The coefficient of corrected relative survival indices is an ordinal scale, where two steps are involved towards its generation. First, the relative survival index was generated, with normalized survival across experiments. Survival days were plotted as dot plots and distribution of survival days for each experiment was determined. Multimodal distribution of survival days grouped into three clusters: susceptible, intermediate, and resistant. A survival index was assigned to each mouse according to which cluster it fell. Indices ranged from 0.25–1, 1.25–2, and 2.25–3 for susceptible, intermediate, and resistant clusters respectively. Relative survival indices were generated for each mouse in each experiment irrespective of its strain, age or weight. Relative survival index data were then subjected to 2-way ANOVA using general linear model (GLM) analyses to generate “Coefficient of corrected relative survival index.” This coefficient is a mean of each strain survival indices, expressing the contribution of each BXD strain towards survival. Covariates that might affect differential sensitivity of different BXD strains to severity of disease were corrected.

## 4. Conclusions

BALB/c and C57BL/6 mice are typically the mouse of choice in MA-EBOV pathogenesis studies with inoculation by the IP route. BALB/c and C57BL/6 mice have been shown not to be susceptible by the SC route and interestingly, were found in this study not to be susceptible to aerosolized MA-EBOV infection. Immunocompromised mice (Stat1 KO, SCID, IFNγ KO, and Perforin KO) were found to be highly susceptible to MA-EBOV infection by the aerosol route. Additionally, Stat 1 KO and SCID mice were also susceptible to “wild-type” EBOV infection by the aerosol route, albeit with a prolonged disease course, particularly for the SCID mice.

B6 and D2 mice were somewhat susceptible to aerosolized MA-EBOV with 0–10% and 0–30% lethality respectively in the two experiments (high and low dose) performed in this study. These two strains are the parent strains of a number of recombinant inbred “BXD” (for B6 × D2) strains. These 80 plus strains are stable, inbred mouse strains for which the recombination sites have been mapped [15,19]. BXD strains have been used to determine gene loci and even genes involved in different disorders (such as alcoholism) and susceptibility and resistance to infectious disease [31,32]. 

We were able to obtain 60 of BXD strains to determine their susceptibility or resistance to aerosolized MA-EBOV. In doing so, five strains were identified that are completely susceptible to a high (approximately >2000 pfu) dose of MA-EBOV. Two of these strains were also highly susceptible to a low dose (approximately 200 pfu) of aerosolized MA-EBOV and had 90 to 100% mortality. Interestingly, in one of these two strains (BXD34), no lung lesions developed in the five mice that were analyzed by histopathology. However, in the strain with 90% lethality (BXD68) at the lower dose of aerosolized MA-EBOV, each of the five mice analyzed by histopathology were found to have areas of necrosis within the lung. Also of note is that there were no signs of disease in eight of 60 BXD strains, which means that these strains were less susceptible than the two parent strains (D2 and B6). 

These studies revealed that genetic differences in BXD mouse strains, and not confounding factors such as age, baseline weight or dose, play a role in susceptibility to MA-EBOV. Several chromosomes and recombination sites have been singled out as probable locations of alleles conferring susceptibility, most notably chromosome 6; however more work will enable mining of specific candidate genes. Interferon response genes are good candidates in conferring susceptibility, and these have not been ruled out. While other pathways could certainly be involved in conferring susceptibility in these strains, the putative QTLs on chromosome 6 that were identified encode at least several proteins involved in the interferon response. For example, on locus rs13478687, FAM40b (also called STRIP2 for striatin-interacting protein 2) forms a protein-protein interaction with SIKE (“suppressor of IKKε), which is a negative regulator for the interferon pathway [33,34]. Another locus, rs3665582 contains transmembrane protein 229A (TMEM229a), which has an interferon binding domain [35]. Furthermore, locus rs3701429 contains the ubiquitin conjugating enzyme (UBE2H), which interacts with interferon-inducible tripartite-motif (TRIM) 56, which is a regulator of double-stranded DNA-mediated type I interferon induction [36]. Chromosome 9 also has such examples; locus rs4227587 encodes the E26 avian leukemia oncogene 1 (ETS1) 5’ domain, which can contribute to up-regulation of the type 1 IFN pathway [37]. Further experiments and analyses of the preliminary QTLs may help to verify and identify regions involved in susceptibility or resistance to filovirus infection. 

The biological basis for the pathology differences observed in the lungs of the two most susceptible BXD strains is also of great interest. BXD68 and BXD21 mice had lung lesions consisting of multifocal congestion, edema, hemorrhage and necrosis. This may be unique to an aerosol route of infection; lung lesions have not been reported in mice infected IP with MA-EBOV. However, multifocal pulmonary congestion, edema, and hemorrhage has recently been reported in interferon receptor-deficient mice that were aerosol-challenged with “wild-type” EBOV; necrosis was apparently not a feature of the lesions in the mice of this latter study ^1^°. In contrast, the BXD34 mice did not have lung lesions. These mice received a slightly higher viral dose than the other animals and they became moribund 1-2 days earlier than the mice in the other two groups. The lack of lung lesions in these mice might have been related to genetic differences in this mouse group and/or the earlier timing of their deaths. The underlying reasons for these differences will be explored in future studies. 

The five BXD strains that were susceptible to airborne MA-EBOV were not susceptible to airborne WT-EBOV. Since the mice lost weight however, it is possible that lethal disease could occur if a higher dose is given or that these mice might be susceptible to WT-EBOV administered by another route. These susceptible BXD mouse strains may also be useful in developing models with other filovirus/route combinations, which have proved difficult thus far. 

There are several similarities between the aerosolized MA-EBOV infection of mice reported here and NHPs infected with filovirus by the aerosol route. Specifically, the lymphocyte death, lesions in the lungs, liver and kidney observed here are also observed in NHPs ^4^. Furthermore, the time at which moribund condition (7–12 days) is reached is also similar for NHPs exposed to filovirus by the aerosol route ^3−5^. Unlike NHPs however, aerosolized MA-EBOV of mice was not associated with evidence of disseminated intravascular coagulopathy (DIC). We conclude that BXD68 and BXD34 are both highly susceptible to aerosolized MA-EBOV infection and either could be used as a model for early stage testing of filovirus countermeasures. 
